# Author Correction: Bridging the literacy gap for surgical consents: an AI-human expert collaborative approach

**DOI:** 10.1038/s41746-024-01099-4

**Published:** 2024-04-12

**Authors:** Rohaid Ali, Ian D. Connolly, Oliver Y. Tang, Fatima N. Mirza, Benjamin Johnston, Hael F. Abdulrazeq, Rachel K. Lim, Paul F. Galamaga, Tiffany J. Libby, Neel R. Sodha, Michael W. Groff, Ziya L. Gokaslan, Albert E. Telfeian, John H. Shin, Wael F. Asaad, James Zou, Curtis E. Doberstein

**Affiliations:** 1 Department of Neurosurgery, Rhode Island Hospital and The Warren Alpert Medical School of Brown University, Providence, RI USA; 2 Norman Prince Neurosciences Institute, Providence, RI USA; 3 Department of Neurosurgery, Massachusetts General Hospital, Boston, MA USA; 4 Department of Dermatology, The Warren Alpert Medical School of Brown University, Providence, RI USA; 5 Department of Neurosurgery, Brigham and Women’s Hospital, Boston, MA USA; 6 Department of Surgery & Division of Cardiothoracic Surgery, Rhode Island Hospital and The Warren Alpert Medical School of Brown University, Providence, RI USA; 7 Ratcliffe Harten Galamaga LLP, Providence, RI USA; 8 Departments of Electrical Engineering, Biomedical Data Science, and Computer Science, Stanford University, Stanford, CA USA; 9 Chan Zuckerberg Biohub, San Francisco, CA USA

**Keywords:** Health care, Medical research, Computational biology and bioinformatics, Health services

Correction to: *npj Digital Medicine* 10.1038/s41746-024-01039-2, published online 8 March 2024

In the original version of this Article, author Rachel K. Lim was inadvertently omitted. The original article has been corrected.

