# Supplementary material for: Bridging the literacy gap for surgical consents: an AI-human expert collaborative approach
Source: NPJ Digit Med. 2024 Mar 8;7:63. doi: 10.1038/s41746-024-01039-2 (PMC10923794; doi:10.1038/s41746-024-01039-2)
Supplement: Supplementary file 1 — Supplementary Information 1 [file 41746_2024_1039_MOESM1_ESM.pdf]

Supplementary Information

Consent #1

I understand the following about the procedure named above:
a. Nature and purpose of procedure (Describe in laymen's terms):
b. Material risks of the procedure include, but are not limited to: DEATH, RESPIRATORY ARREST, CARDIAC ARREST, BRAIN DAMAGE, DISFIGURING SCAR, PARAPLEGIA OR QUADRIPLEGIA, PARALYSIS OR PARTIAL PARALYSIS, LOSS OR LOSS OF FUNCTION OF ANY LIMB OR ORGAN, SEVERE LOSS OF BLOOD, ALLERGIC REACTION AND INFECTION.
These are material risks of any procedure. Other risks and/or the potential side effects of this procedure include, but may not be limited to:
c. Likelihood of success: Good, Fair, Poor, Unknown because:
d. Practical alternatives to procedure:
e. Prognosis if procedure rejected: Good, Fair, Poor, Unknown because:
f. If applicable, and as discussed with my physician/surgeon, I temporarily suspend my consent to any Do Not Resuscitate (DNR) or Do Not Intubate (DNI) order during the procedure and recovery period, unless indicated otherwise:
2. The nature and purpose of the procedure identified above have been explained to me, including the potential benefits and side effects of the procedure. I understand the practical alternatives to the procedure and their risks and I hereby consent to the performance of this procedure by __________________________ and/or any assistants who may be present. I also consent to the administration of anesthesia, (including but not limited to general anesthesia, spinal anesthesia, and/or major regional anesthesia), intravenous procedural sedation,
and/or the intravenous or intraductal injection (or administration by any other route) of a contrast material, as determined by my physician/surgeon or his/her qualified designee. I understand that anesthesia, sedation, and/or contrast material will be administered by or under the direction and supervision of ______
understand that some or all of the healthcare professionals performing services as part of my procedures may be independent contractors who are not employees or agents of ______. I understand that independent contractors are responsible for their own actions and that ____are not liable for the acts or omissions of any such independent contractors.
3. I realize that during the procedure, the physician/surgeon or his/her qualified designee, may become aware of conditions which were not apparent before the start of the procedure, or may determine that additional or different operations or procedures are necessary or appropriate. I therefore authorize the above-named physician/surgeon, or his/her qualified designee, and/or any assistants who may be present, to perform additional or different operations or procedures the physician/surgeon, or his/her qualified designee, deems necessary or advisable; so long as these additional procedures do not conflict with my stated DNR or DNI code status as indicated above.
4. I acknowledge and agree that any tissue, organ, specimen, member or implant, removed or severed from my body during the procedure described above, may be retained, preserved, analyzed, and/or disposed of, or may otherwise be used for any lawful purpose, including medical education and teaching, by____
5. I acknowledge that the physician/surgeon, or his/her designee, may photograph, videotape or otherwise make recordings of me or my image before, during, or after this procedure for purposes related to care, treatment and/or medical education.
6. I understand that this consent form will be valid for 30 days, unless I have signed this consent form in conjunction with an admission to the hospital, in which case this consent will be valid for 30 days from the date of admission or for the duration of my hospitalization, whichever is greater.
7. I understand that the physician/surgeon/anesthesiologist or other qualified healthcare professional will be present for the key portions of my procedure. However, because he/she may be coordinating various procedures which overlap with my procedure, I further understand that he/she may not be present during some non-key elements of my procedure, which will be covered by another qualified healthcare professional member of the procedural team.
8. I acknowledge that no guarantees have been made concerning the outcome of the surgical or medical treatment, and I realize that the practice of medicine and surgery is not an exact science. I have read all of the above information, and I have been given the chance to ask any questions, and all of my questions have been answered to my satisfaction.
I REQUEST AND CONSENT TO THE PERFORMANCE OF THE PROCEDURE AS OUTLINED ABOVE

GPT-4 Consent #1

I know the following about the procedure mentioned above:
a. What the procedure is and why it's done (Explain in simple terms):
b. The main risks of the procedure can include, but are not limited to: DEATH, BREATHING STOPPAGE, HEART STOPPAGE, BRAIN DAMAGE, UGLY SCARS, PARALYSIS OF HALF OR ALL BODY, LOSS OF USE OF A LIMB OR ORGAN, HEAVY BLOOD LOSS, ALLERGIC REACTION, AND INFECTION. These risks can happen with any procedure. There might be other risks or side effects from this specific procedure, including but not limited to:
c. Chances of the procedure working: Good, Fair, Poor, or Unknown because:
d. other options instead of the procedure:
e. What could happen if I don't get the procedure: Good, Fair, Poor, or Unknown because:
f. If it applies, I agree with my doctor to temporarily not follow any Do Not Resuscitate (DNR) or Do Not Intubate (DNI) order during the procedure and recovery period, unless otherwise stated:

The procedure and its purpose have been explained to me, including the possible benefits and side effects. I understand the other options and their risks, and I agree to have this procedure done by ___________________________ and any helpers who may be there. I also agree to receive anesthesia, sedation, and/or contrast material as decided by my doctor or their qualified helper. I understand that the people doing the procedure might not all work for the same company or hospital, and that the hospital isn't responsible for their actions.
I know that during the procedure, the doctor or their helper might find out new information or decide that more or different steps are needed. I give permission for the doctor and any helpers to do any extra or different steps they think are needed, as long as it doesn't go against any DNR or DNI orders I have.
I agree that any tissue, organ, sample, body part, or implant taken out during the procedure can be kept, studied, thrown away, or used for teaching by the hospital, clinic, or other medical groups.
I understand that the doctor or their helper might take photos, videos, or recordings of me before, during, or after the procedure to help with my care or for teaching.
I know that this form is good for 30 days, or for 30 days after I go into the hospital if I'm already there.
I understand that the doctor or another qualified person will be there for the important parts of my procedure. But the doctor might not be there for some less important parts, and another qualified person will take their place.
I know that no one can promise how the procedure will turn out, and that medicine and surgery can be unpredictable. I've read all the information above and had the chance to ask questions, and I'm happy with the answers I got. I agree to have the procedure as described above.

Consent #2

I, ____, (Name of Patient or Designated Decision
Maker) have been informed that the surgery, procedure, or treatment to be performed is to:
___________ (site)_____________(side)__________________, the
purpose of which is to _______.

II. Documentation of Informed Consent

X On Form __Attached Office Note __Progress Notes __H&P

3. III.

I understand that the potential benefits and outcomes of the proposed surgery, procedure, or treatment include but are not limited to return to active lifestyle.

I understand that the potential risks and complications associated with the surgery, procedure, or treatment include but are not limited to bleeding / infection/ blood clot, injury to nerve/vessel/muscle/ tendon, fracture, dislocation, foot drop, leg length discrepancy, failure of surgery, loss of limb or life, allergy to implant or failure of hardware, need for further surgery.

Alternatives to the proposed surgeries, procedures, and treatments for my condition including the option of no treatment have been discussed with me. These include but are not limited to:
no surgery

Serial Procedures – I understand that I will receive a series of the same treatments over a time period not to exceed 180 days.

From ___/___/___to___/___/___

IV. Specific to Surgery

1. It has been explained to me that sometimes during surgery, it is discovered that additional surgery is needed. If, in the opinion of
the doctor who is operating, I need such additional surgery, I permit the doctor to proceed.
2. I consent to being photographer or filmed during the course of m operation or treatment. I understand that and that the photographs/films will be used for educational and research purposes only and that my name will not be placed on the photographs/films used for educational and research purposes. I understand the photographs intended for my medical record will be identified by my name and may not be released or used without my expressed agreement. NA
_____________I do not want my photographs/films released or used for educational purposes.
3. I authorize the physicians and the _____ Health System to preserve for scientific research, or teaching purposes, or to dispose of any tissues, body parts, or organs removed as a necessary part of my care according to hospital policy, with the following exceptions: ________________
(Send copy of any exceptions to Department of Pathology).
4. I understand that, at the request of my physician, a vendor or medical equipment representative may be present during the performance of my procedure. Presence shall be limited to providing information for coordination of treatment and technical expertise on the use and operation of the vendor's device under the supervision of my physician.

GPT-4 Consent #2

I, __________________ (Patient Name or Decision Maker), understand that the medical procedure, surgery, or treatment to be done is:

II. Proof of Informed Consent

X On Form __Attached Office Note __Progress Notes __H&P

III.

I know that the possible benefits and results of the suggested surgery, procedure, or treatment may include, but are not limited to:

I know that the possible risks and problems linked to the surgery, procedure, or treatment may include, but are not limited to:

Other options for my condition, including not having any treatment, have been talked about with me. These options may include, but are not limited to:

no surgery

Series of Procedures – I understand that I will get the same treatments several times over a period of no more than 180 days.

From _________to________

IV. About Surgery

I have been told that sometimes during surgery, more surgery is found to be needed. If the doctor doing the surgery thinks I need more surgery; I allow the doctor to go ahead.

I agree to be photographed or filmed during my surgery or treatment. I know that the photos/films will only be used for learning and research, and my name won't be on the photos/films used for these purposes. I know that photos meant for my medical records will have my name and can't be shared or used without my permission. NA _____________I don't want my photos/films shared or used for learning purposes.

I let the doctors and the _____ Health System keep for research or teaching, or throw away any tissues, body parts, or organs taken out during my care, following hospital rules, except for these: ________________ (send a copy of any exceptions to the Department of Pathology).

I know that, if my doctor asks, a seller or medical equipment expert might be there during my procedure. They will only be there to give information about the treatment and help with using the equipment under my doctor's watch.

Consent-4 #3

Acknowledgment of Consent for Surgical or Other Procedure

You have the right to be informed about the surgical or other procedure(s) which your physician recommends so that you can make an informed decision whether to undergo the procedure(s). The purpose of this form is to provide written acknowledgment of your consent.

I voluntarily authorize this physician, as well as his/her partner, assistant, or designee to perform the surgical or other procedure(s) described below:

Primary procedure(s) to be performed (if within OR):

My condition and the above procedure(s) have been described to me. Alternative treatments for my condition and the risks of alternative treatment or no treatment at all have been explained. I understand that during my surgery or procedure(s) my physician may decide that there are additional surgeries or procedures which may be required, and I consent to those surgeries or procedures which in my physicians' professional judgement are necessary. The potential need for blood transfusions was explained where appropriate, along with a discussion of the potential risks, benefits, and alternatives to transfusion.

Anesthesia

If a healthcare worker receives an occupational exposure to my blood or body fluids during the procedure and I am unable to provide consent at the time of the exposure, I consent to an immediate HIV blood test so that the healthcare worker can receive prompt attention if necessary.

I understand that genetic or diagnostic testing may be done on tissue, fluids or organs removed during surgery. I understand that this is a teaching facility, and that the Hospital may use remaining portions of tissue, fluid or organs that are not sent for genetic or diagnostic testing for teaching, research, or scientific purposes, or may otherwise dispose of them. I understand that in this process, the Hospital will take appropriate steps to protect my confidentiality.

I understand that audio/video equipment may be in use during all or part of my procedure for clinical care or quality monitoring. No recordings made during my procedure will be used for educational or performance improvement purposes without my prior completion of an additional consent.

I also acknowledge that residents and assistants designated by my physician may participate in the procedure and there may be other observers or vendors present.

I understand that I am under no obligation to proceed with the surgery or procedure until all requested information has been provided and all my questions have been answered to my satisfaction. I acknowledge that this has been done.

GPT-4 Consent #3

Permission for Surgery or Other Treatment

You have the right to know about the surgery or other treatments your doctor suggests so you can decide if you want to have them. This form is to show you agree in writing.

I give permission for this doctor and their partner, helper, or someone they choose to do the surgery or other treatment(s) listed below:

Main treatment(s) to be done (if in the operating room):

My health problem and the treatment(s) listed above have been explained to me. I have been told about other ways to treat my problem and the risks of not getting treated or choosing a different treatment. I understand that during my surgery or treatment, my doctor might decide I need more surgeries or treatments. I agree to let my doctor do any other surgeries or treatments they think I need. If I might need a blood transfusion, they have told me about the possible risks, benefits, and other options.

Anesthesia

If a healthcare worker is exposed to my blood or body fluids during the treatment and I can't give permission at that time, I agree to have an HIV blood test right away. This will help the healthcare worker get the care they need if necessary.

I understand that tests might be done on tissue, fluids, or organs taken out during surgery. I know that this is a teaching hospital and leftover tissue, fluid, or organs not used for testing might be used for teaching, research, or science, or might be thrown away. I know that the hospital will try to keep my information private during this process.

I understand that audio/video equipment might be used during some or all of my treatment for patient care or to check the quality. No recordings made during my treatment will be used for teaching or improving how things are done without me agreeing first in writing.

I also know that medical residents and helpers chosen by my doctor might be part of the treatment, and there might be other people watching or vendors in the room.

I know that I don't have to go through with the surgery or treatment until I get all the information I want and all my questions are answered in a way that makes me happy. I agree that this has been done.

Consent #4

The recommended procedure, intervention, or treatment is:
The reasons for this recommended procedure, intervention, or treatment have been discussed with me and include:
The expected benefits of this procedure, intervention, or treatment have been discussed with me and include:
The potential risks of this procedure, intervention, or treatment, if any, have been discussed with me and may include:
Other options to this procedure, intervention, or treatment, if any, have been discussed with me and include:
If blood or blood products are needed, a clinician has discussed with me, and I agree to the following:
I give permission for the administration of blood or blood products (whole blood, red blood cells, platelets, plasma, or cryoprecipitate) while in the hospital, both during and following this procedure, if the treating medical team believes it is medically necessary. I understand that donated blood and blood products is tested for infections and compatibility. I am aware of the related risks with using blood or blood products, as described on this form.
I have reviewed with a clinician and agree to the following:
I acknowledge that I was given the opportunity to discuss and ask questions about the recommended procedure(s), intervention(s), or treatment(s), including the expected benefit(s) and potential risk(s).
I was informed if there are other options to this procedure, intervention or treatment and explained the possible risks of me saying "no" to the procedure, intervention, or treatment.
I give permission to my Primary Provider to change the plan during the recommended procedure(s), intervention(s), or treatment(s), if in their professional judgment they find it is medically necessary to do so.
I understand that I can change my mind about the recommended procedure, intervention, or treatment, and I agree that if I plan to do so I must tell my Primary Provider before the procedure starts.
I give permission to my Primary Provider to get help from others (including but not limited to physicians, advance practice registered nurses, physician assistants, residents, and fellows) during the procedure. I understand anyone actively participating who is not listed on this form will be documented in my medical record, and available to me on the patient portal once the care team finishes their note about the procedure.
I give permission for using and testing any tissue, implant, or related biological material that is removed during the procedure, intervention, or treatment for research (without identification), or for treatment, quality assurance, and training. I also give permission for my health information, including results from diagnostic testing, to be combined with other patient information in a clinical reference database for treatment, quality assurance, or training to compare results with other patients, including those with same or similar conditions.
I give permission for other clinical staff (including but not limited to physicians, advance practice registered
nurses, physician assistants, residents, and fellows) to watch for training purposes.
I give permission for photos, videos, and/or recordings to be taken for training purposes (without identification, treatment, or health care operations.)

Parent/Guardian and/or Patient Signature
I have been given an opportunity to ask questions and my questions have been answered to my satisfaction. I have reviewed this form and give permission for the procedure, intervention, or treatment, according to the terms described in this form.

GPT-4 Consent #4

We talked about a suggested treatment or procedure and why it is recommended. We also discussed the benefits of this treatment, possible risks, and other options available.
If I need blood or blood products during the procedure, the medical team will use them. I understand that donated blood is tested for infections and compatibility. I know there are risks with using blood products, as explained on this form.
I talked with a doctor about the treatment, its benefits and risks, other options, and what happens if I don't agree to it. I can change my mind, but I will let my doctor know before the procedure starts. My doctor can change the plan during the procedure if needed. Other medical professionals may help during the procedure or watch for training purposes. Photos or recordings may be taken for training or treatment purposes (without my name).
Other providers who will be in the room during the procedure, if known when I sign this form, are listed below:
Provider Name/Position
I had a chance to ask questions and they were answered. I agree to the treatment as described in this form.
Parent/Guardian Signature Name Relationship Time Date
Patient Signature Name Time Date
Providers involved in the treatment talked with me about it and answered my questions.
Provider Obtaining Consent Signature Name Credentials Time Date
Witness/Interpreter Signature Name Credentials Time Date
Was an Interpreter used: Yes/No/Parent/Guardian and/or Patient Refused.
Interpretation Services: Interpreter Tablet Application Language line
Why would blood or blood products be needed? What are the risks?

- Blood and blood products can help with oxygen supply or bleeding issues.
- Donated blood is tested for diseases like HIV, Hepatitis B and C, West Nile, and Syphilis. The risk of getting a disease from donated blood is low. Patients may have reactions like allergies, fevers, fluid complications, lung injury, or reactions from incompatible blood. These reactions are usually treatable, and patients recover quickly. However, in rare cases, patients may die from blood transfusion complications.
- If you refuse blood or blood products, some services may be limited during or after the treatment. Separate consents or agreements may be needed if you refuse blood products.

We've discussed and agreed upon the procedure or treatment. By signing this form, you give permission for the medical team to proceed with the suggested treatment plan.

You've been informed about potential risks, benefits, and alternative options. Remember that you have the right to change your mind and can inform your doctor at any time before the procedure starts.
It is essential to understand that other medical professionals may be present during the procedure to assist or observe for educational purposes. Additionally, photos, videos, or recordings may be taken without your identification for training or treatment purposes.
If you have any further questions or concerns, feel free to ask your medical team. They are here to help and provide you with the best possible care. It's crucial to be informed and comfortable with your decision before moving forward with the procedure or treatment.
Once you've reviewed and understand all the information, please sign below to confirm your consent:
Parent/Guardian Signature: ________________________
Name:________________________ Relationship: ________________________ Time:_______ Date: _______
Patient Signature: ________________________ Name: ________________________
Time: _______ Date: _______
It is important that you have had an opportunity to discuss the procedure, intervention, or treatment with your providers. They will ensure that you understand the information, answer any questions and address your concerns. Remember that an interpreter may be used if needed, and there are various interpretation services available to help facilitate communication between you and your medical team.
By signing this form, you acknowledge that you've been given a chance to ask questions, and your questions have been answered to your satisfaction. You're giving permission for the procedure, intervention, or treatment according to the terms described in this form.

Consent #5

We talked about a suggested treatment or procedure and why it is recommended. We also discussed the benefits of this treatment, possible risks, and other options available. If I need blood or blood products during the procedure, the medical team will use them. I understand that donated blood is tested for infections and compatibility. I know there are risks with using blood products, as explained on this form.

I talked with a doctor about the treatment, its benefits and risks, other options, and what happens if I don't agree to it. I can change my mind, but I will let my doctor know before the procedure starts. My doctor can change the plan during the procedure if needed. Other medical professionals may help during the procedure or watch for training purposes. Photos or recordings may be taken for training or treatment purposes (without my name).

Other providers who will be in the room during the procedure, if known when I sign this form,
are listed below:

Provider Name Position Provider Name Position

I had a chance to ask questions and they were answered. I agree to the treatment as described in this form.

Parent/Guardian Signature Name Relationship Time Date

Patient Signature Name Time Date

Providers involved in the treatment talked with me about it and answered my questions.

Provider Obtaining Consent Signature Name Credentials Time Date

Primary Provider's Signature Name Credentials Time Date

Witness/Interpreter Signature Name Credentials Time Date

Was an Interpreter used: Yes/No Parent/Guardian and/or Patient Refused?

Interpretation Services: Interpreter Tablet Application Language line

Why would blood or blood products be needed? What are the risks?

- Blood and blood products can help with oxygen supply or bleeding issues.
- Donated blood is tested for diseases like HIV, Hepatitis B and C, West Nile, and Syphilis. The risk of getting a disease from donated blood is low. Patients may have reactions like allergies, fevers, fluid complications, lung injury, or reactions from incompatible blood. These reactions are usually treatable, and patients recover quickly. However, in rare cases, patients may die from blood transfusion complications.
- If you refuse blood or blood products, some services may be limited during or after the treatment. Separate consents or agreements may be needed if you refuse blood products.

We've discussed and agreed upon the procedure or treatment. By signing this form, you give permission for the medical team to proceed with the suggested treatment plan.

You've been informed about potential risks, benefits, and alternative options. Remember that you have the right to change your mind and can inform your doctor at any time before the procedure starts.

It is essential to understand that other medical professionals may be present during the procedure to assist or observe for educational purposes. Additionally, photos, videos, or recordings may be taken without your identification for training or treatment purposes.

If you have any further questions or concerns, feel free to ask your medical team. They are here to help and provide you with the best possible care. It's crucial to be informed and comfortable with your decision before moving forward with the procedure or treatment.

Once you've reviewed and understand all the information, please sign below to confirm your consent:

Parent/Guardian Signature: ________________________ Name:_______ Date: _______

Patient Signature: ________________________ Name: ________________________

Time: _______ Date: _______

It is important that you have had an opportunity to discuss the procedure, intervention,
or treatment with your providers. They will ensure that you understand the information,
answer any questions and address your concerns.

Remember that an interpreter may be used if needed, and there are various interpretation services available to help facilitate communication between you and your medical team.

By signing this form, you acknowledge that you've been given a chance to ask questions, and your questions have been answered to your satisfaction. You're giving permission for the procedure, intervention, or treatment according to the terms described in this form.

GPT-4 Consent #5

We talked about a suggested treatment or procedure and why it is recommended. We also discussed the benefits of this treatment, possible risks, and other options available.
If I need blood or blood products during the procedure, the medical team will use them. I understand that donated blood is tested for infections and compatibility. I know there are risks with using blood products, as explained on this form.
I talked with a doctor about the treatment, its benefits and risks, other options, and what happens if I don't agree to it. I can change my mind, but I will let my doctor know before the procedure starts. My doctor can change the plan during the procedure if needed. Other medical professionals may help during the procedure or watch for training purposes. Photos or recordings may be taken for training or treatment purposes (without my name).
Other providers who will be in the room during the procedure, if known when I sign this form, are listed below:
Provider Name Position Provider Name Position
I had a chance to ask questions and they were answered. I agree to the treatment as described in this form.
Parent/Guardian Signature Name Relationship Time Date
Patient Signature Name Time Date
Providers involved in the treatment talked with me about it and answered my questions.
Provider Obtaining Consent Signature Name Credentials Time Date
Primary Provider's Signature Name Credentials Time Date
Witness/Interpreter Signature Name Credentials Time Date
Was an Interpreter used: Yes/No Parent/Guardian and/or Patient Refused?
Interpretation Services: Interpreter Tablet Application Language line
Why would blood or blood products be needed? What are the risks?
Blood and blood products can help with oxygen supply or bleeding issues.
Donated blood is tested for diseases like HIV, Hepatitis B and C, West Nile, and Syphilis. The risk of getting a disease from donated blood is low. Patients may have reactions like allergies, fevers, fluid complications, lung injury, or reactions from incompatible blood. These reactions are usually treatable, and patients recover quickly. However, in rare cases, patients may die from blood transfusion complications.
If you refuse blood or blood products, some services may be limited during or after the treatment. Separate consents or agreements may be needed if you refuse blood products.
We've discussed and agreed upon the procedure or treatment. By signing this form, you give permission for the medical team to proceed with the suggested treatment plan.

You've been informed about potential risks, benefits, and alternative options. Remember that you have the right to change your mind and can inform your doctor at any time before the procedure starts.
It is essential to understand that other medical professionals may be present during the procedure to assist or observe for educational purposes. Additionally, photos, videos, or recordings may be taken without your identification for training or treatment purposes. If you have any further questions or concerns, feel free to ask your medical team. They are here to help and provide you with the best possible care. It's crucial to be informed and comfortable with your decision before moving forward with the procedure or treatment.
Once you've reviewed and understand all the information, please sign below to confirm your consent:
Parent/Guardian Signature: ________________________ Name:________________________ Time: _______ Date: _______
Patient Signature: ________________________ Name: ________________________
Time: _______ Date: _______
It is important that you have had an opportunity to discuss the procedure, intervention, or treatment with your providers. They will ensure that you understand the information, answer any questions and address your concerns.
Remember that an interpreter may be used if needed, and there are various interpretation services available to help facilitate communication between you and your medical team. By signing this form, you acknowledge that you've been given a chance to ask questions, and your questions have been answered to your satisfaction. You're giving permission for the procedure, intervention, or treatment according to the terms described in this form.

Consent #6

I have been informed of 1) the potential risks and benefits of the procedure(s); and 2) the risks and benefits of the alternatives, including the consequences of not having the procedure(s). I am aware that the practice of medicine and surgery is not an exact science, and I acknowledge that no guarantees have been made to me concerning the results of the proposed treatment(s) or procedure(s).

Further I am aware that there are possible risks, such as loss of blood, infection or pain that may accompany any surgical, diagnostic, or therapeutic procedure. The following additional risks were explained to me:

If procedural sedation will be used during this procedure, I understand that this sedation has risks. My physician has discussed the use of procedural sedation. The risks include but are not limited to slower breathing and low blood pressure that may require treatment.

I understand that a potential risk or complication of the procedure is the loss of blood. I understand that I may require blood products during the procedure or in the post-procedure period. If I refuse blood products, I will complete a separate release for blood-free treatment form.

I understand that one or more healthcare industry professionals (technical representatives for medical equipment and device companies) or observers may be present during this procedure for advisory or observational purposes only.

The hospital may photograph, videotape, or record my procedure/surgery for educational, research, quality and other healthcare operations purposes. Any information used for these purposes will not identify me.

I understand that blood or other samples removed during this procedure may later be disposed of by Hospital. These materials also may be used by the Hospital, its partners, or affiliates for research, education and other activities that support hospital's mission.

A team of medical professionals will work together to perform my procedure/surgery. The role and involvement of the senior attending in my procedure has been discussed with me, including that he/she may join the procedure after the opening of the surgical site or may leave during the closing of the surgical site, and may need to step away during non-critical portions of the procedure. The roles of additional practitioners involved in the procedure, indicated below, have also been explained to me. I understand that other medical professionals may be involved in the procedure who are not listed below. The name of those practitioners will be shared with me after the procedure.

I have had a chance to ask questions about the risks, benefits, side effects, likelihood of achieving the goals of this procedure, and other approaches. All my questions were answered to my satisfaction, and I give permission to have the procedure.

GPT-4 Consent #6

I have been told about 1) the possible good and bad things that could happen from the procedure(s); and 2) the good and bad things that could happen from other choices, including what might happen if I don't get the procedure(s). I know that doctors and surgeons can't always be sure how things will turn out, and I understand that no one has promised me that the results of the treatment(s) or procedure(s) will be perfect. I also know that there might be problems, like losing blood, getting an infection, or feeling pain, that could happen with any surgery, test, or treatment. The doctor also told me about these other risks:

If I need medicine to make me sleepy during the procedure, I know that there are risks. My doctor talked to me about using this medicine. Some risks include breathing more slowly and having low blood pressure that might need treatment. I know that losing blood is another possible problem from the procedure. I understand that I might need blood products during the procedure or after it. If I say no to blood products, I will fill out a separate form for blood-free treatment.

I know that one or more healthcare industry professionals (people who work for medical equipment and device companies) or observers might be there during the procedure to give advice or just watch.

The hospital can take pictures, video, or record my procedure/surgery to help with teaching, research, and other healthcare purposes. They won't use my name if they do this.

I know that blood or other samples taken during this procedure may later be thrown away by the hospital. The hospital might also use these materials for research, education, and other activities that help the hospital do its job.

A team of medical professionals will work together to do my procedure/surgery. The senior attending's role and how much they will be involved in my procedure have been explained to me. This includes that they might join after the surgery has started or leave before it is finished, and they might need to step away during parts that aren't as important. The roles of other people involved in the procedure have also been explained to me. I know that other medical professionals might help with the procedure but aren't listed here. Their names will be given to me after the procedure.

I had a chance to ask questions about the risks, benefits, side effects, how likely it is that the procedure will help, and other options. All my questions were answered, and I am okay with having the procedure.

Consent #7

I understand that any operation or procedure may involve the risk of an unsuccessful result or complication, including but not limited to bleeding, infection, nerve/nervous system damage, injury to organs/structures, or even death from both known and unforeseen causes. My physician has explained to me the nature and purpose of the operation or procedure along with the risks, benefits and alternatives of the procedure to my satisfaction. In addition, the risks and benefits of these alternatives, and the risks of having no treatment have been explained to me. I have had the opportunity to ask questions and have received all the information I desire about the operation or procedure. Except in an emergency, I understand that an operation or procedure is not performed until I have had the opportunity to receive this information and have given my consent.

In an emergency, I understand there may be different or further procedures required if the physician believes they are necessary and I consent to such procedures.

I understand that the administration of anesthesia and/or moderate sedation may be necessary to assure safety and comfort during the procedure and I consent to the administration if indicated. I understand that certain risks and complications may be associated with the use of anesthesia and/or moderate sedation and the physician has discussed these risks with me prior to the procedure.

I understand that the operation or procedure may involve the use of a Food and Drug Administration (FDA) approved drug or device for a purpose not approved by the FDA. I understand that other medical care will not be withheld if I decide to withhold or withdraw my consent to this proposed treatment.

I understand that ____ is an educational institution and as part of the medical education and training program, postgraduate fellows, residents, medical students, surgical assistants, approved health care practitioners and visiting professors may observe care, and if appropriately trained, participate in aspects of the operation or procedure under the supervision of my physician.

If applicable, my physician has informed me of the potential for an overlapping surgery. I understand that my physician will be present during key or critical portions of my procedure or surgery and in some circumstances, my physician may participate in another operation following the key or critical portions of my surgery. In this circumstance, my physician will be immediately available or will ensure another qualified physician is immediately available.

I understand that if the procedure involves specialized equipment or medical device(s}, the Manufacturer's representative(s) may be present during the procedure to assist in the selection or calibration of the equipment or device(s) and in the related treatment.

I consent to the hospital's use and/or disposal, at its discretion, of any blood, bodily fluid, member, organ, or other tissue removed or obtained during the operation or procedure for research that may be conducted by ___, unaffiliated academic or commercial third parties If allowed under legal requirements and relevant policies.

I consent to the taking of pictures, videotapes or other electronic reproductions of my medical or surgical condition or treatment, and the use of the pictures or videotapes for treatment or internal or external activities consistent with _____ mission, such as education and research, conducted in accordance with Hospital policies.

I authorize NAME OF PHYSICIAN / PRACTITIONER performing procedure

to perform the following OPERATION OR PROCEDURE: [Spell out all words. Do not abbreviate. Identify side/level of procedure to be performed if applicable.]

By my signature below, I confirm that:

I have read, understand, and agree to the above; My physician has provided me with information to make a fully informed decision to undergo the operation or procedure documented above; and

I consent to the performance of the operation or procedure.

GPT-4 Consent #7

Please read this whole form carefully before you sign it.

I know that any operation or procedure might have risks. These risks include things like bleeding, infection, damage to nerves or organs, or even death. These problems can come from things we know about and things we don't.

My doctor has told me what the operation or procedure is for and what it will do. They have also told me about the good and bad things that might happen and other choices I have. They have told me about what might happen if I don't get any treatment. I've had the chance to ask questions and get all the information I want. I know that I need to say it's okay before they can do the operation or procedure, except in an emergency.

In an emergency, I know that the doctor might need to do more things to help me. I'm okay with them doing these things.

I know that I might need to be given anesthesia or sedation to help me feel better during the procedure. I'm okay with this. My doctor has talked to me about the risks that come with anesthesia or sedation.

I know that sometimes the doctor might use a drug or device that is approved by the FDA for a different reason. I also know that if I don't want to do this, they will still give me other medical care.

I know that _____ is a place where people learn. This means that other doctors, students, and people in training might watch my care or help with my procedure. My doctor will be watching them.

If needed, my doctor has told me that they might need to be in another surgery during my procedure. They will only do this after the important parts of my surgery are done. If they're not there, another doctor will be ready to help.

I know that there might be special equipment or devices used during the procedure. People who know how to use these things might be there to help.

I'm okay with the hospital using or getting rid of any blood, fluids, or tissue that they take out during my procedure. They might use these things for research.

I'm okay with the hospital taking pictures or videos of my treatment. They can use these for things like teaching or research.

I give my permission for

NAME OF DOCTOR doing the procedure, to do the OPERATION OR PROCEDURE: [Write out all words. Don't use abbreviations. Say which side or level the procedure is on if needed.]

By signing below, I agree that:

I've read and understand everything above.

My doctor has given me enough information to decide if I want the operation or procedure.

I'm okay with having the operation or procedure.

If I give my permission by phone, someone will sign for me. They will write their information below.

DATE TIME SIGNATURE (Patient / Person who can make decisions for me)

PRINT NAME

If someone helped translate for me, they will write their information below:

RELATIONSHIP TO PATIENT

Print ____ person who translated name Video or Phone Translator ID# Language

TELEPHONE PERMISSION: (Only if the person who can make decisions for me can't be there to sign this form).

DATE TIME SIGNATURE NAME OF WITNESS/HOSPITAL WORKER PRINT NAME

*The witness/hospital worker (like a resident, nurse, or PA) must:

listen to the phone call where the person making decisions for me says it's okay for the operation or procedure; and say that the person making decisions for me gave their permission.

Consent #8

I have been advised that this procedure may have potential benefits, risks and side effects including but not limited to I have been advised of the alternatives, benefits and side effects related to the alternatives. I have been advised of the likelihood of achieving my goals and any potential problems that might occur during recuperation.
I understand that the practice of medicine is not an exact science and I acknowledge that I have received no guarantees about the benefits or results of this treatment. I have read this entire document and understand it. I have been given the opportunity to ask questions and my questions have been answered to my satisfaction.

I verify that I have explained the procedure, relevant risks, benefits and alternatives, benefits and side effects related to alternatives, potential problems during recuperative phase, treatment and services, and possible results of not receiving care.
I consent to the administration of anesthesia and related drugs, as deemed necessary by the staff members from Anesthesiology.
I understand that unforeseen complications or conditions may arise during this procedure, and I consent to any additional procedures that the physician(s) may deem advisable in their professional judgment.
I understand that portions of the operation/procedure may be photographed or videotaped. I understand that every attempt will be made to conceal my identity. I understand that some of these photograph/videotapes may be used for teaching and may not be maintained or be a part of my medical record. I also understand that photographs/videotapes to plan, monitor or document my treatment may be part of my medical record.
I understand that residents, medical, nursing and allied health students/trainees may be present during the procedure and they may observe or assist in my care, under the direction of my surgeon and/or other hospital staff members.
I understand that a sales/clinical representative may be present during the procedure, but may not participate in the procedure. I impose no specific limitations or restrictions on my treatment unless written below:

GPT-4 consent #8

I've been told that this procedure could have benefits, risks, and side effects. I know about other options, their benefits, and side effects too. I've been told how likely it is that I'll reach my goals and what problems might happen while I'm healing.

I know that medicine isn't perfect, and I haven't been promised any specific results from this treatment. I've read this whole paper and understand it. I've had the chance to ask questions and am happy with the answers.

I confirm that I've explained the procedure, its risks and benefits, other options, and what might happen during recovery, as well as the possible results if I don't get care. I agree to getting anesthesia and related drugs, as the staff from _____ think is needed. I know that unexpected problems might come up during the procedure, and I agree to any extra steps the doctors think are best.

I know that parts of the operation might be photographed or recorded on video. They'll try to hide my identity, and these photos or videos might be used for teaching but might not be kept or be part of my medical record. Photos or videos used to plan, watch, or document my treatment could be part of my medical record.

I know that medical, nursing, and other health students might be there during the procedure and could watch or help with my care, under the direction of my surgeon and hospital staff. I know that a sales or clinical representative might be there during the procedure, but they won't take part in it. I don't have any special limits or restrictions on my treatment, unless I write them down here:

Consent #9

I agree to have Dr. [name]______________________________("Surgeon"), and any associates or assistants selected by Surgeon to do the following operation(s) or procedure(s) ("Procedure") on the patient named above ("Patient"):

____
____
____

2. All Procedures have risks, including bleeding, infection, and even death.
I have been informed of such risks, as well as the nature of the Procedure, the likelihood of achieving goals, the expected benefits, or effects of such Procedure, including side effects and potential problems that might occur during recuperation, and the reasonable alternative methods of treatment and their risks and benefits. I have also been informed of the consequences of declining the Procedure and the reasonable alternatives.
3. I agree to have additional procedures if, during the Procedure, my Surgeon decides they are needed for reasons not known before the Procedure. I also authorize my Surgeon to provide or arrange for the provision of additional services, as necessary or advisable, including but not limited to, pathology and radiology services.
4. _______ is a teaching institution. Resident physicians and students may work with the surgeon. Resident physicians may do part of the surgery. The Surgeon will decide at the time of the surgery which residents will take part. What they are allowed to do will depend upon their skill and the Patient's condition. Residents will be under the supervision of the Surgeon. There are times when an attending Surgeon will oversee the care provided by teams in two operating rooms simultaneously, defined as concurrent staffing. The Surgeon or an attending designee will be present for all the critical parts of the procedure/ surgery. The Surgeon may be out of the operating room for some or all of the surgical tasks done by residents if the Surgeon decides it is safe to do so.
5. Trained, licensed people who are not physicians may do part of the surgery or administer the anesthesia. They will be only doing things allowed by the hospital and by law.
6. My Surgeon may have healthcare industry representatives or other visitors present in the operating room for technical support related to my treatment, and I authorize those individuals to be present.
I DO NOT authorize those individuals to be present.
7. My Surgeon does not have any financial or research interest in the Procedure, (including but not limited to a consulting or services agreement, or receipt of research funding) with a vendor of any products or devices used in the procedure, other than his/her professional fees, unless noted below: __________(initial if applicable) My Surgeon has informed me that he/she has a financial relationship with a vendor of products or devices used in the procedure.

CONSENT FOR SURGERY OR SPECIAL DIAGNOSTIC or THERAPEUTIC PROCEDURE(S)

8. _____ may keep, use or dispose of anything removed during the Procedure("Specimens"). Specimens may be used for research. I do not own the Specimens, or data derived from Specimens, and have no right to any research or research product using or derived from the Specimens. Specimen includes, but is not limited to, any tissues, organs, bones, bodily fluids, or medical devices.
9. I have the right to consent to or to refuse any proposed operation or procedure, including the Procedure, at any time prior to its performance. I am aware that the practice of medicine and surgery is not an exact science, and no guarantee has been made as to the results of the Procedure or any cure. I also understand that the explanations that I have received may not be exhaustive or all-inclusive and that other more remote risks may be involved. However, the information that I have received is sufficient for me to consent to the Procedure. I have had a full opportunity to ask questions concerning my condition, the Procedure, the risks, and the alternatives. All of the questions that I have asked have been answered to my satisfaction.
10.I understand that if an implantable device is used, information regarding the device and my Social Security Number may be reported to the device manufacturer, if requested, and as required by Federal law.
1) I have read and understood the information contained here;
2) I have been informed about the Procedure and the potential risks, benefits, alternatives
and the risk of those alternatives;
3) I authorize and consent to the performance of the Procedure as described; and
4) I authorize and direct that any Specimen removed during the Procedure is to be handled

as indicated above.

GPT-4 Consent #9

I agree to let Dr. [name]______________________________("Doctor") and their team do the surgery or treatment ("Treatment") listed above on the patient named above ("Patient"):
____
____
____

2. All Treatments have risks, like bleeding, infection, and even death. I know about these risks and other details, like what the Treatment is, if it will likely work, its benefits, side effects, and possible problems during recovery. I also know about other ways to treat my issue and their risks and benefits. I understand what might happen if I don't get the Treatment and my other choices.
3. If the Doctor thinks I need more procedures during the Treatment, I agree to have them. I also let the Doctor get other services for me if needed, like tests and imaging.
4. _____ is a teaching hospital. Trainee doctors and students may help the Doctor. They might do part of the surgery under the Doctor's watch. Sometimes, another senior doctor may watch over the team in two different operating rooms at the same time. The Doctor, or someone they choose, will be there for the most important parts of the surgery. The Doctor may leave the room if they think it's safe for the trainees to continue without them.
Trained, licensed non-doctors may do part of the surgery or give me anesthesia. They will only do what the hospital and the law allows.
My doctor may have people from the healthcare industry or visitors in the operating room for technical help during my Treatment, and _____I let them be there. ___ I DON'T let them be there.
My Doctor doesn't have money or research interests in the Treatment, like deals with a company that makes products used in the surgery, except for their professional fees, unless noted below: __________ (initial if true) My Doctor told me they have a financial relationship with a company that makes products used in the surgery.
CONSENT FOR SURGERY OR SPECIAL DIAGNOSTIC or TREATMENT 8. ______ can keep, use, or throw away anything taken out during the Treatment ("Samples"). Samples can be used for research. I don't own the Samples or any research or products that come from them. A Sample can be things like tissue, organs, bones, fluids, or medical devices.
I can say yes or no to any operation or treatment, including this one, before it starts. I know that medicine and surgery don't always work perfectly, and no one promised me a perfect result or cure. I understand that I might not know everything about the risks, but I know enough to agree to the Treatment. I had a chance to ask questions about my issue, the Treatment, the risks, and other options. I got answers to all my questions.
If a device is put in my body, information about the device and my Social Security Number might be shared with the device maker, if asked and as required by law.
I read and understood the information here;
I know about the Treatment, its risks, benefits, other choices, and their risks;
I agree to the Treatment as explained; and
I let any Samples taken during the Treatment be used as described above.

Consent #10

I have reviewed this copy of "Surgical/Procedure Risks" and had the opportunity to talk with my provider about the benefits, potential outcomes, and likelihood of achieving the goals of the planned procedure(s); risks and possible complications; alternatives to the procedure, including the risks and benefits associated with no treatment; and, possible problems that may occur during my recuperation. I have been thoroughly informed, and had all of my questions answered.

I understand that my diagnosis is: ____

I have consented to the following by, or under the supervision of: ___________________________:

1) Planned Procedure(s) ____
____
____
2) Any other procedures necessary to address conditions that are discovered during the procedure(s) listed above;
3) Disposal or retention of any tissues removed during the procedure;
4) Pictures or videotape being taken during the procedure so long as my identity is not revealed;
5) Observers for medical education being present in the operating room.

GPT-4 Consent #10

I have looked over the "Surgery and Treatment Risks" document and talked with my doctor about the benefits, possible results, and chances of reaching the goals of the planned treatment(s); risks and possible problems; other options, including the risks and benefits of not getting treated; and any issues that might come up during my recovery. I have been given all the information I need and all of my questions have been answered. I understand that my medical problem is: ____

I agree to the following, done by or supervised by: _____________________________________:

Planned treatment(s) ____

____

____

Any extra treatments needed to deal with issues found during the treatments listed above;

Throwing away or keeping any body parts taken out during the treatment;

Photos or videos being made during the treatment as long as no one can tell it's me;

People watching for learning purposes being in the surgery room.

Consent #11

I give permission to my health care professionals (who are listed on the back of this form as the performing provider), to do the procedure(s) listed on the back of this form, with anesthesia and/or sedation if that is needed. Anesthesia or sedation medicine will be given by the health care professional, anesthesiologist, or other trained health care staff who work under appropriate supervision.
I understand that the attending physician may need to perform other urgent procedures due to an emergency that may occur while I am sedated or otherwise not able to give consent. The attending physician or designee will talk with my legally authorized representative if possible.
I understand that the attending physician may choose assistants, including other health care professionals and residents (physicians who have finished medical school, but are getting more training), to be part of the team performing my procedure.
The assistants may suture; harvest grafts; dissect, remove or alter tissue; implant devices; or do other tasks that the attending physician has deemed appropriate. If known, the attending physician has discussed with me whether there will be assistants and whom she expects the assistants to be. I understand that during the procedure, the attending physician may need to choose different assistants or have them do different tasks. I understand that for some kinds of medical equipment used during procedures, a representative from the equipment manufacturer may be there, doing things like providing consultation or running checks on the equipment.
I understand the attending physician may participate in care involving overlapping procedures or oversee other urgent medical responsibilities and may not be always present in the room. During this time, the skilled team of assistants may perform portions of my procedure which the attending physician has determined they are proficient to perform. If an overlap is anticipated or planned, I will be notified prior to surgery. The attending physician will be present for the key and critical portions of the procedure, and either he/she or a designated attending physician will be always available to the procedure team.
The hospital or health care professional will dispose of any removed tissues or parts.
I understand what procedure(s) will be done. I have been told about the risks and benefits. I have been told about other treatment choices and about their risks and benefits, including not having the procedure. I have been told about what results to expect, which includes information about the chances for the expected results. I know that results cannot be guaranteed. I have been told about potential problems that may occur during recuperation.
I understand that there are risks for all kinds of surgery and for "invasive procedures" (procedures where a blood vessel, body cavity, or other internal tissue is entered with a needle, tube, or similar device). These risks, which can be serious, include bleeding, infection, and damage to nearby tissues, vessels, nerves, or organs. They may even result in paralysis, cardiac arrest, brain damage, and/or death.
I have received this added detailed information and/or patient information materials about the procedure(s):
Print added information or title of information materials
I understand whether I will receive either anesthesia or sedation medicine, or both. I have been told about my choices for anesthesia and sedation and about their risks and benefits. I have been told about side effects of the medicine(s) and problems they may cause with recovery.
I understand that anesthesia and sedation medicines used for procedures involve risks. These risks can be serious. They may include damage to vital organs such as the brain, heart, lungs, liver, and kidneys. They may even result in paralysis, cardiac arrest, brain damage, and/or death.
I understand that the anesthesia equipment may damage my teeth or cause other dental damage.
1 understand that nerve damage may occur from how anesthesia equipment is placed or how my body must be positioned during a procedure.
I understand that I am free to refuse consent to any proposed procedure.
BLOOD: I have been told whether I am having a procedure where blood or blood components (products) may need to be used (also known as transfused). If I am having this kind of procedure, I have been told about side effects, risks, and other choices about transfusion, including not getting a transfusion.
I give permission to receive blood and/or blood components if the health care team decides it is needed. I understand that use of blood and blood components involves risks. The risks may include reactions, including allergic reactions, fever, hives, lung injury, and in rare cases, infectious diseases such as hepatitis and HIV/AIDS. I know that the blood bank screens donors and matches blood for transfusions to help lower risks.

OR please initial refuse (or partially refuse) permission for blood and blood components. (You will be asked to sign another form, Form _____).
Giving Consent: By signing below, I confirm that I have read the sections above and that I have had 1) each item explained to me; 2) a chance to ask questions; and 3) all my questions answered.

GPT-4 Consent #11

I allow my health care workers (named on the back of this form) to perform the procedure(s) listed on the back of this form. They can use anesthesia or sedation if needed. The health care worker, anesthesiologist, or other trained staff will give me the anesthesia or sedation.

I know that the main doctor might need to do other urgent procedures if there's an emergency while I'm sedated or can't give permission. They will try to talk to my legal representative if possible.

I know that the main doctor might choose helpers, like other health care workers and medical residents, to be part of the team doing my procedure. These helpers might do tasks like stitching, getting grafts, working with tissue, putting in devices, or other tasks the main doctor thinks are right. The main doctor has told me about these helpers and who they might be. I know that during the procedure, the main doctor might need to pick different helpers or give them different tasks. I also know that sometimes a person from a medical equipment company might be there to help or check the equipment.

I know that the main doctor might have to do other important medical tasks and might not always be in the room. During this time, the skilled helpers might do parts of my procedure that the main doctor thinks they can do well. If there's a planned overlap, I will be told before surgery. The main doctor will be there for the most important parts of the procedure, and they or another main doctor will always be available to the team.

The hospital or health care worker will get rid of any tissue or parts that are taken out.

I know what procedure(s) will be done, the risks, benefits, and other treatment options. This includes not having the procedure. I know what results to expect and the chances of getting those results. I know that no one can promise results. I know about possible problems during recovery.
I know that all surgeries and "invasive procedures" have risks. These risks can be serious and include bleeding, infection, and damage to nearby tissues, blood vessels, nerves, or organs. They can even cause paralysis, heart problems, brain damage, or death.

I got more information and/or materials about the procedure(s): (Write down the extra information or title of materials)

I know if I will get anesthesia or sedation medicine, or both. I know about the choices, risks, and benefits. I know about the side effects and any problems they might cause during recovery.

I know that anesthesia and sedation medicine have risks. These can be serious and include damage to important organs like the brain, heart, lungs, liver, and kidneys. They can even cause paralysis, heart problems, brain damage, or death.
I know that the anesthesia equipment might damage my teeth or cause other dental problems.

I know that nerve damage might happen from how the anesthesia equipment is placed or how my body is positioned during the procedure.
I know that I can say no to any proposed procedure.

BLOOD: I know if I'm having a procedure where I might need blood or blood products. If I am, I know about side effects, risks, and other options, including not getting a transfusion.
I allow the health care team to give me blood and/or blood products if needed. I know there are risks, like reactions, fever, lung injury, and very rarely, diseases like hepatitis and HIV/AIDS. I know that the blood bank tests donors and matches blood to lower risks.

OR initial to refuse (or partly refuse) blood and blood products. (You will be asked to sign another form, Form ____).

Giving Permission
By signing below, I confirm that I have read the sections above and that I have had 1) each item explained to me; 2) a chance to ask questions; and 3) all of my questions answered.

Consent #12

After discussing other options, including no treatment, with the responsible practitioner or his/her delegated representative, I give permission to perform the following operation, procedures, or treatment (list name of description or operation, indicate applicable level, side, or site)
I understand that this procedure is for purposes of diagnosis and/or treatment of (describe reasons for procedure)
I give permission to my responsible practitioner to do whatever may be necessary if there is a complication or unforeseen condition during my procedure.
My responsible practitioner has explained to me in a way that I understand: the nature and purpose of the procedure, the potential benefits and risks and possible side effects of the procedure both during it and during recuperation, including bleeding, infection, accidental injury of other body parts, failure to permanently improve my condition or death, as well as the potential risks and benefits of the medications that may be administered to me as part of the procedure, and the alternative to the procedure and their potential risks and benefits, including the option of not having the procedure. I understand that the other complications may occur, including but not limited to:
I understand the purpose and potential benefits of the procedure in relation to my goals. My responsible practitioner has explained to me what results to expect, and the chances of achieving them. I understand that no promises or guarantees have been made or can be made about the results of the procedure.
I agree to have anesthesia as necessary to perform the procedure. I understand that if an anesthesiologist is to be involved, he or she will speak to me about the risks of anesthesia in more detail and I may be asked to sign a separate anesthesia or sedation consent form.
I give permission to the hospital and or its departments to examine and keep tissue, blood, body parts, or fluids removed from my body during the procedure to aid in diagnosis and treatment, after which they may be used for scientific research or teaching by appropriate persons. If these things are used for signs or teaching, identity will not be disclosed. I will no longer own or have any rights to these things regardless of how they may be used.
If the procedure listed above involves the implantation or transplantation of tissue from a human or animal source my responsible practitioner has described to me the risks and benefits of, and alternatives to, receiving this product.
I understand that some of the system hospitals are teaching hospitals. Doctors or other health practitioners who are members of the care team and are in training may help my practitioner with the procedure. I understand that these trainees are supervised by qualified staff and the responsible practitioner will be present at all important times during the procedure. I also understand that the associate, surgical assistance and other non physicians or trainees may assist my responsible practitioner or perform parts of the procedure under the responsible practitioner supervision as permitted by law and hospital policy. This includes compliance with the overlapping surgery policy which ensures that the attending surgeon will be present for the critical key portions of my case and that an alternative attending physician will be designated should the need arise. If others who are not hospital staff will be present in the operating room, the responsible practitioner has spoken with me about this. I understand that a representative of an equipment vendor or a visitor may be present in the procedure area and that if that occurs, any visitor or vendor will comply with any applicable policy regarding observers in the operating room or other procedural area.
I give permission to the hospital and the above named practitioner to photograph and or visual recorder or display the procedure for medical, scientific, or educational purposes. I understand that I will not be identified by those not involved in my care unless a separate consent is signed.
I understand that my responsible practitioner may deem it necessary for me to have a good blood transfusion during or after the procedure. I understand what a blood transfusion is, the procedure used, the benefits of receiving a transfusion and the risks involved. Benefits include better oxygen to delivery to all parts of my body and treating or decreasing the risks of bleeding. The risks include fever chills and allergic reactions which are generally mild and transient, on rare occasions major transfusion reactions occur such as rapid breakdown of blood cells and acute lung or kidney injury, and rarely bacterial viral or other infections such as hepatitis, hepatitis C, human immunodeficiency virus and other pathogens. I understand these risks exist, although screening and testing of blood donors and their blood is performed to minimize these risks. My questions regarding alternatives have been addressed by the responsible practitioner and relation to my specific circumstances.

In the event a healthcare worker is exposed to my blood or body fluids in connection with my procedure, or during my hospital stay, I agree to the collection and testing of my blood for HIV. I have read this form or had it read to

me. I have had an opportunity to ask questions and to consider my decisions. All of my questions have been answered to my satisfaction.

GPT-4 Consent #12

After talking about other choices, like not having any treatment, with my main doctor or someone they chose, I say it's okay to do this surgery or treatment (list what it's called, where it will happen, and any other details).

I know this is being done to find out what's wrong and/or to help me get better (describe why it's needed).

I let my main doctor do what they need to do if something unexpected happens during my treatment.

My doctor has told me about: what the surgery or treatment is for, the good and bad things that might happen during and after it (like bleeding, infection, hurting other parts of my body, not getting better, or even dying), what medicines I might get and what they do, other ways to treat my problem, and what could happen if I don't have the treatment at all. I know other problems could come up, including:

I know why I'm having this and what good things might happen. My doctor told me what results I might get and how likely they are. I know that no one can promise me how it will turn out.

I'm okay with getting put to sleep (anesthesia) if I need to. I know that if there's a special doctor for this, they'll talk to me more about the risks and I might have to sign another form.

I let the hospital and its people look at and keep any tissue, blood, body parts, or fluids they take out of me during the treatment. They can use these things to learn more and teach others, but they won't tell anyone who I am. I won't own these things or have any rights to them after that.

If my treatment needs something from a human or animal, my doctor has talked to me about the good and bad things that could happen and other choices I have.

I know that some hospitals are places where doctors and other health workers learn. Some of these learners might help my doctor with my treatment. I know they are watched by people who know what they're doing and that my main doctor will be there when it matters most. Other people might help my doctor or do parts of the treatment under their watch, as the law and hospital rules allow. If people who don't work at the hospital are in the room where the treatment happens, my doctor has told me about it. I know that if someone is there to watch, they will follow the rules.

I let the hospital and my doctor take pictures or videos of my treatment for learning or teaching reasons. I know they won't say who I am unless I sign another form.

I know my doctor might think I need a blood transfusion during or after the treatment. I know what it is, how it's done, the good things about it (like getting more oxygen and less chance of bleeding), and the bad things that could happen. Some risks include fever, chills, or allergies, which usually aren't bad and don't last long. In rare cases, there might be more serious problems, like my body breaking down blood cells fast, or damage to my lungs or kidneys. It's very rare, but sometimes people get infections like hepatitis, hepatitis C, HIV, and others. I know that the blood is tested to make sure it's safe. My doctor has answered my questions about other choices for my situation.

If someone working on my healthcare gets in contact with my blood or body fluids during my treatment or hospital stay, I agree to let them test my blood for HIV. I've read this form or had someone read it to me. I've had a chance to ask questions and think about my decision. All my questions have been answered and I'm happy with the answers.

Consent #13

I, the undersigned, consent to the following operation(s) and / or procedure(s);
to be performed by Dr. _____and his / her associates and assistants, as indicated below, with knowledge that the attending physician will have primary responsibility for my care specific to the stated procedure.
1 understand that different or additional procedures that my physician considers emergent and necessary may be required during the course of the operation(s) / procedure(s) described above and I consent to such procedures.
I understand that physicians who are fellows or residents (resident physicians), may also be involved in the procedure(s), including performing one or more significant task. I further understand that if resident physicians are involved:
They will perform portions of the procedure(s) based on their level of competence;
It will be decided at the time of the procedure(s) which resident physicians will participate and their manner of participation, taking into account the following factors: 1) my condition, 2) the availability of resident physicians with the necessary competence, and 3) the knowledge of the supervising physician of the resident physicians' skill sets;
Any resident physicians performing significant tasks will be under the supervision of their supervising physician, though based on the resident physicians' level of competence, the supervising physician may not be physically present in the same room for some or all of the tasks performed by resident physicians.
I have had the opportunity to ask any questions that I have regarding resident physician involvement.
I understand that my procedure may overlap with another procedure that my physician is scheduled to perform.
I understand that this means my physician will be present in the operating room during the critical parts of my surgery, but may not be present for my entire procedure. I understand that my physician or another qualified attending physician will be immediately available should the need arise during my procedure. My physician has answered all of my questions about overlapping operations and I give my consent.
I understand that certain significant tasks may be performed by qualified medical practitioners who are not physicians, acting within their scope of practice as permitted by State law and their clinical privileges granted by the hospital.
Dr. has explained to me the nature and purpose of each operations) and / or procedure(s) as well as the substantial risks and possible complications involved, the benefits and the medically reasonable alternative methods of treatment.
The SUBSTANTIAL RISKS include but are not limited to (add additional risks as indicated):
[perforation and / or injury to adjacent blood vessels, nerves and / or organs, bleeding, infection]
The POTENTIAL BENEFIT(S) include but are not limited to:
The MEDICALLY REASONABLE ALTERNATIVE(S) are:
I understand and consent to _____ disposing of any tissue, parts or organs that are removed during the operation(s) and / or procedure(s), in accordance with its usual practice.
I understand that the information I have received about risks is not exhaustive and there may be other, more remote risks.
I have had the opportunity to ask questions regarding the proposed procedure(s) and all my questions have been answered to my satisfaction.
I have read or have had read to me, this Operative / Invasive Procedure Informed Consent form.
I have had explained to me, and I understand the potential benefits and drawbacks, potential problems related to recuperation, the likelihood of success, the possible results of non-treatment, and any medically reasonable alternatives.
I have received no guarantees from anyone regarding the results that may be obtained.
I know the relationship, if any, of my physician or other practitioner, to any teaching facility involved in my care.
CONSENT
I do hereby consent to the above described operation(s) and / or procedure(s).
Patient Signature
Date
Time_

By placing my initials at the end of this sentence, I also consent to having observers present during my procedure, in accordance with my physician's approval and hospital policy.
Initials
Patient Printed Name
Staff Witness Signature
Staff Witness Printed Name

GPT-4 Consent #13

I, the person signing below, agree to the following surgery or treatment. Dr. ______ and their team will do the surgery or treatment listed below. The main doctor will be in charge of my care for this surgery or treatment.

I know that other unexpected things might need to be done during the surgery or treatment. If my doctor thinks it's needed, I agree to let them do those things.
I know that doctors-in-training (called fellows or residents) might help with the surgery or treatment. They will do tasks based on what they know and can do. The main doctor will decide which doctors-in-training will help and how they will help. They will think about my health, the skills of the doctors-in-training, and how well the main doctor knows their skills. Doctors-in-training will have a supervisor, but the supervisor might not be in the room all the time.

I have had a chance to ask questions about doctors-in-training helping with my surgery or treatment.

I know that my surgery might happen at the same time as another surgery my doctor has to do. I know my doctor will be there for the most important parts of my surgery but might not be there the whole time. If needed, my doctor or another doctor will be nearby to help. My doctor has answered my questions about this, and I agree to it.

I know that some tasks might be done by trained medical workers who aren't doctors. They will do things they are allowed to do by law and the hospital.

Dr. ______ has told me what the surgery or treatment is for, the big risks and possible problems, the benefits, and other ways to treat my health issue.

There are big risks, like (list any extra risks):
holes or injuries to nearby blood vessels, nerves, or organs
bleeding
infection
The possible benefits are:
(list benefits)

Other medical choices are:
(list choices)

I agree to let ______ throw away any tissue, parts, or organs taken out during the surgery or treatment. They will do this the usual way.

I know that the list of risks is not complete and there might be other risks.
I have had a chance to ask questions about the surgery or treatment. My questions have been answered in a way that I am happy with.
I have read or had someone read to me this Surgery/Treatment Permission form. I understand the possible benefits and problems, how I might feel after, if it will likely work, what could happen if I don't get treated, and other medical choices.
Nobody has promised me any results.

I know if my doctor or another medical worker is part of a teaching facility that is helping with my care.

I agree to the surgery or treatment listed above.
Patient Signature: ______________________
Date: _______________ Time: _______________

By putting my initials here (_____), I agree to let people watch my surgery or treatment if my doctor says it's okay and it follows the hospital rules.
Initials: _____
Patient Printed Name: ______________________
Staff Witness Signature: ______________________
Staff Witness Printed Name: ______________________

Consent #14

I have been asked to read all of the information contained in this consent form and to consent to the procedure described below on behalf of (fill in name of patient) or myself. I have been told that I should ask questions about anything that I do not understand. (If the decision-maker signing this form is not the patient, references to "I," "my" or "me" should be read as if referring to "the patient.") I understand that the information in this consent form, in addition to discussions with my physicians and any other written materials they may provide, is intended to help me make an informed decision whether to voluntarily undergo the identified procedure.
Diagnosis: I understand that after being examined, treated, and having studies reviewed, I have been diagnosed as having:
Recommended Procedure: I understand that my physician(s) have recommended that I undergo a procedure known as
I acknowledge that my physician(s) or physician representative has described the recommended procedure to my satisfaction including the risks and benefits of the procedure, alternative treatments, the risks and benefits of the alternative treatments, the
likelihood of me achieving my goals; any potential problems that might occur during recuperation and the likely medical results should I decide not to undergo the recommended procedure. I have also been told that there are risks that may occur with any surgery even in healthy patients. These risks include, but are not limited to bleeding, which may require the use of blood or blood products, injury to adjacent organs including the spleen, stroke, heart attack, infection, death, cardiac arrest, brain and nerve damage (including paralysis, loss of function, and coma).
Additional risks:
If needed, blood and/or blood products have the following general risks: reactions resulting in itching, rash, fever, chills, headache, or shock; respiratory distress (shortness of breath); kidney damage; systemic bacterial infection; exposure to blood borne viruses including hepatitis (an inflammatory disease affecting the liver) and Human Immunodeficiency Virus (HIV, the virus that causes AIDS); and death. Alternatives to transfusion include the use of devices that filter and return blood lost in surgery to me or by providing medications that boost my blood count prior to an elective procedure. Bleeding and/or severe anemia could put my life in danger or cause permanent brain damage. I understand that substitutes for blood or plasma might not work well enough. Blood and/or blood products might offer the only chance to preserve my life. I refuse the transfusion of blood and/or blood products and understand that I will be asked to sign a separate form entitled, Release from Liability for the Refusal of Blood Transfusion.
If my procedure is to be performed in an Ambulatory Surgical Facility (ASF), the comparative risks, benefits and alternatives associated with performing the procedure in the ASF instead of a hospital have been fully explained to me.
I understand the hospital may require that all jewelry and/or body piercing hardware be removed prior to surgery.
Teaching Facility and Overlapping Surgeries: I understand that the facility is a teaching facility. The health care team may include residents, fellows, students, and skilled healthcare professionals. Credentialed team members may perform all or parts of my procedure under the supervision and guidance of my physician(s). My attending physician may also be caring for one other patient during my surgery, but remains responsible to me and will perform or be present for the key portions of the procedure. If unanticipated circumstances require my surgeon to be unavailable during my surgery, another qualified surgeon will promptly come to the operating room. Representatives of medical device companies may be present to provide devices, and observe and advise on their use. Who will participate and in what manner will be decided at the time of the procedure and will depend on the availability of individuals with the necessary expertise and on my medical condition.
If an accidental exposure to my blood or body fluids occurs to staff during the surgery or procedure I agree to blood tests for hepatitis B, hepatitis C and HIV. I understand that the physician(s) or others may choose to photograph, televise, film or otherwise record all or any portion of my procedure for medical, scientific or educational purposes. I consent to the photographing, televising, filming or other forms of recording of the procedure(s) to be performed, including appropriate portions of my body, body functions or sounds, provided my identity is not revealed. I understand and agree that 1) any photographs, films, or other audio or visual recordings created will be the sole property of the facility: and 2) the facility or any appropriate staff member may edit, preserve, or destroy all or any part of the photographs, films, or other audio or visual recordings. Such recordings are not part of the medical record and I understand I cannot obtain a copy. I authorize the disposal or retention, preservation, testing, or use for scientific, educational or other purposes of all or any portion of specimens, tissues, body parts, or other things, including prostheses and medical/surgical appliances, that may be removed from my body. I understand that if any medical device, as defined by federal regulations, is implanted in a patient's body, the facility is required by law to report to the manufacturer the name, address and social security number of the patient and the description and identity of the device.
MY SIGNATURE BELOW ACKNOWLEDGES THAT:
1. I have read (or had read to me), understand and agree to the statements set forth in this consent form.
2. A physician has explained to me all information referred to in this consent form. I have had an opportunity to ask questions and my questions have been answered to my satisfaction, including any question I have about the potential use of blood and/or blood products and any risks regarding their use.
3. All statements requiring completion were filled in before I signed.
4. No guarantees or assurances concerning the results of the procedure(s) have been made.
5. I am signing this consent voluntarily. I am not signing due to any coercion or other influence.
6. I hereby consent and authorize Dr. ____________________________(my physician(s)) and/or those associates, assistants and other health care providers designated by my physician(s) to perform the recommended procedure described above. I understand that during the course of the procedure, conditions may become apparent that require my physicians or their designees to take steps or perform additional procedures that they believe are medically
necessary to achieve the desired benefits or for my well-being. I authorize and request my physician(s) or their designees to perform whatever medical acts or additional procedures they, in the exercise of their sole professional judgment, deem reasonable and necessary, and I waive any obligation on their part to stop or delay the continuation of my procedure in order to obtain additional consent if I am unable to give additional consent at that time.

GPT-4 Consent #14

I need to read and agree to everything in this consent form for the procedure for either (fill in patient's name) or myself. If I don't understand something, I should ask questions. (If you're signing for someone else, "I," "my," and "me" should be read as if referring to the patient.) The consent form, conversations with my doctors, and any written materials they give me will help me decide if I want to go through with the procedure.

Diagnosis: After being checked, treated, and having tests looked at, my diagnosis is:

Recommended Procedure: My doctors suggest I have a procedure called:

My doctor(s) or their representative has explained the procedure, its risks and benefits, other treatment options, risks and benefits of those options, my chances of reaching my goals, possible problems during recovery, and what could happen if I don't get the procedure. I know there are risks with any surgery, even for healthy people. These risks include, but aren't limited to, bleeding, needing blood or blood products, injury to nearby organs (like the spleen), stroke, heart attack, infection, death, cardiac arrest, and brain and nerve damage (including paralysis, loss of function, and coma).

Additional risks:

If needed, blood and/or blood products have risks like reactions causing itching, rash, fever, chills, headache, or shock; trouble breathing; kidney damage; bacterial infection; exposure to viruses like hepatitis (liver inflammation) and HIV (which causes AIDS); and death. I can avoid transfusion with devices that filter and return lost blood during surgery or by taking medicine to raise my blood count before a planned procedure. Bleeding and/or severe anemia can be life-threatening or cause permanent brain damage. Blood substitutes might not work well enough, and blood or blood products might be the only way to save my life. If I refuse a blood transfusion, I will sign a separate form called Release from Liability for the Refusal of Blood Transfusion.

If my procedure happens at an Ambulatory Surgical Facility (ASF), they will explain the risks, benefits, and alternatives of doing the procedure there instead of a hospital.

I understand that I may need to remove jewelry or body piercings before surgery.

Teaching Facility and Overlapping Surgeries: The facility is a teaching place. The healthcare team might include residents, fellows, students, and skilled professionals. Qualified team members might do all or parts of my procedure with my doctor(s) supervising. My main doctor might also care for another patient during my surgery but will still be responsible for me and be there for key parts of the procedure. If my surgeon can't be there during my surgery, another qualified surgeon will come to the operating room. Medical device company reps might be there to provide devices and give advice on using them. Who takes part and how depends on who's available with the right skills and on my medical condition.

If staff are accidentally exposed to my blood or body fluids during surgery, I agree to blood tests for hepatitis B, hepatitis C, and HIV.

I understand my doctor(s) or others might take photos, videos, or recordings of my procedure for medical, scientific, or educational purposes. I'm okay with that, as long as they don't reveal my identity. The facility will own these recordings, and they can edit, keep, or destroy them as they choose. The recordings aren't part of my medical record, and I can't get a copy. I let the facility keep or use any specimens, tissues, body parts, or other things removed from my body for scientific, educational, or other purposes. If a medical device is put in my body, the facility must give the manufacturer my name, address, social security number, and device details by law.

MY SIGNATURE BELOW MEANS THAT:

I've read (or had read to me), understand, and agree with everything in this consent form.

A doctor has explained all the information in this consent form to me. I've had a chance to ask questions, and they've answered them to my satisfaction, including any questions about blood and/or blood products and their risks. All the required statements were filled in before I signed.

No one has promised or guaranteed the results of the procedure(s). I'm signing this consent form willingly, not because someone forced or pressured me. I give permission to Dr. ___________________________(my doctor(s)) and/or their associates, assistants, and other healthcare providers they choose to perform the procedure explained above. I understand that during the procedure, my doctors or their designees might find conditions that need more steps or extra procedures for my benefit or well-being. I let my doctor(s) or their designees do whatever medical acts or extra procedures they think are necessary. I don't expect them to stop or delay my procedure to get more consent if I can't give it at that time.

Consent #15

I give my permission for the procedure(s) listed below to be done on ___________ by or under the direction of __________________.

2. This medical/surgical procedure is called:______ In plain language, the procedure is described as:______

3. An intimate exam (pelvic, prostate, or rectal exam): IS NOT indicated during this procedure. WILL/WILL NOT be performed during this procedure.

4. Expected benefits of this procedure have been explained to me by the operating practitioner (additional comments optional):

5. Expected risks and complications of this procedure, including the potential problems in the recuperative period and the likelihood of achieving my treatment goals, have been explained to me by the operating practitioner. (additional comments optional):

6. Alternatives to this procedure, if they exist, have been explained to me as well as the consequences of no treatment. I have been informed that there may be unforeseen problems during any procedure. I have been informed that there are no guarantees or warranties about the benefits of any procedure, or about the risks and complications that I may sustain.

7. I agree to the use of anesthesia / sedation as explained to me for this procedure. If I have an anesthesia care provider, I have been informed that such person will obtain separate anesthesia consent.

8. If there is an emergency, or if something unexpected happens during the procedure, I have been informed and accept that my physicians will take whatever emergency measures are needed to help me, including additional procedures, anesthesia, or transfusion of blood components.

9. Physicians other than the operating practitioner, including but not necessarily limited to residents, will be performing important tasks related to the surgery, in accordance with the hospital's policies and, in the case of residents, based on their skill set and under the supervision of the responsible practitioner.

Authorization for Medical/Surgical Procedure

Printed name: ______________________________________

Interpreter Required for all patients or medical decision-makers except those whose preferred language is English or non-verbal:

If an intimate exam is for training purposes:

Pursuant to PA 22-33, I have been informed that an intimate exam is not clinically indicated as part of this medical/surgical procedure, but that a medical student, resident or fellow will be performing an intimate exam exclusively for training purposes and I give my consent to the performance of the intimate exam.

Signed by ______________________________________ Date: ____________ Time:

10. Qualified medical practitioners who are not physicians who will perform important parts of the surgery or administer anesthesia will be performing only tasks that are within their scope of practice, as determined under State law and regulation, and for which they have been granted privileges by the hospital.

11. At the discretion of my practitioner(s), there may be students present and /or health care industry representatives who provide technical expertise or who may program implantable devices (e.g., pacemakers).

12. The hospital will oversee the disposition of any specimen(s) or tissue removed during this procedure in an appropriate manner; this may include retaining some of my tissue for research purposes, provided that all links to my identity have been destroyed and cannot be reconstructed.

I have had the informed consent discussion with my operating practitioner and I give my consent for this procedure. I have had the opportunity to ask questions of the operating practitioner that have been answered to my satisfaction.

GPT-4 Consent #15

I give permission for the following procedure(s) to be done on ___________________________ by or under the direction of ________________________________. 2. The medical or surgical procedure is called: ___________________.

In simple words, the procedure is described as: ____

A personal exam (pelvic, prostate, or rectal exam): WON'T be done during this procedure. WILL/WON'T be done during this procedure.

The doctor has told me the expected benefits of this procedure (more details can be added): ____

The doctor has explained the expected risks and problems that may happen during recovery and the chance of reaching my treatment goals (more details can be added): ____

The doctor has explained other options for this procedure, if there are any, as well as what could happen if I don't get treated. I know that unexpected problems can happen during any procedure. I've been told that there are no promises or guarantees about the benefits, risks, or complications.

I agree to the use of anesthesia or sedation for this procedure as explained to me. If I have someone giving me anesthesia, I've been told they will get separate permission.

If there's an emergency or something unexpected during the procedure, I know and accept that my doctors will do whatever is needed to help me, like extra procedures, anesthesia, or giving me blood.

Other doctors, not just the main one, may help with important parts of the surgery, following the hospital's rules and under the supervision of the main doctor.

Permission for Medical/Surgical Procedure Printed name: _______________________________________

____. Interpreter needed for all patients or decision-makers except those who prefer English or can't speak:

If a personal exam is for training: Under PA 22-33, I've been told that a personal exam isn't needed for this medical/surgical procedure, but a medical student, resident, or fellow will do it for training. I give my permission for the personal exam. Signed by _______________________________________ Date: ____________ Time:

Medical professionals who aren't doctors will only do parts of the surgery or give anesthesia that they're allowed to do under state laws and rules, and that the hospital lets them do.

My doctor(s) may have students or health care industry reps who give technical help or work with devices (like pacemakers) present.

The hospital will handle any samples or tissue removed during the procedure correctly; this may include using some of my tissue for research, as long as my identity is removed and can't be found.

I've talked with my main doctor about this procedure, and I give my permission. I've had the chance to ask questions and the doctor has answered them to my satisfaction.

Supplementary Figure 1A: Consent for deep brain stimulation for Parkinson's Disease

Spatz et al. consent form abstraction and scoring system:

Prompt:

Write a consent form for an awake subthalamic nucleus deep brain stimulator electrode implantation with microelectrode recordings and intra-operative test stimulation for Parkinson disease. Include both quantitative and qualitative probability of risks. Ensure it is written at the average American reading level.

Title: Permission Form for Brain Surgery to Help Parkinson's Disease

Patient Name: ______________________________
Date of Birth: ______________________________

Dear Patient,

This permission form explains the brain surgery that may help your Parkinson's disease. Please read this form carefully and ask any questions if something is unclear. Your doctor will gladly answer your questions or concerns.

Purpose of the Surgery
The surgery aims to treat Parkinson's disease symptoms like shaking, stiffness, and trouble moving. A device called a deep brain stimulator (DBS) will be put in a part of your brain called the subthalamic nucleus. This device sends electrical signals to help reduce your symptoms and make your life better.

Description of the Surgery
You will be awake but relaxed during the surgery. The doctor will make a small cut on your head and a tiny hole in your skull. A thin wire, called a microelectrode, will be put into the subthalamic nucleus. This wire records brain activity, helping the doctor find the best place for the DBS device.

After finding the best place, the doctor will put in the DBS device and test it to make sure it works. If the test goes well, the doctor will secure the device and close the cuts.

Risks and Complications
All surgeries have risks and problems. The risks for this brain surgery are:
Infection: 1-3% chance (rare)
Bleeding or blood clot: 1-2% chance (rare)
Seizure: 0.5-1% chance (very rare)
Stroke: 0.1-0.5% chance (extremely rare)
Device not working right: 1-4% chance (rare)
Needing another surgery: 2-7% chance (uncommon)
These risks are low, but some can cause severe problems or even death.

Benefits of the Surgery
The possible benefits of this surgery include:
Less shaking, stiffness, and trouble moving
Better life quality
Less need for medicines
Remember that results are different for everyone, and not all patients will see the same improvement.

Other Options
Other choices instead of this brain surgery include:
Medicines: Changing your current medicines may help your symptoms.
Physical, job, and speech therapy: These therapies can help you manage symptoms and improve daily life.

Patient Permission

By signing this form, you agree that you have read and understood the information given. You give your permission for the brain surgery to help your Parkinson's disease. You also agree that you talked about the risks, benefits, and other options with your doctor.

Patient Signature: _____________________

Date: _____________________

Doctor Signature: _____________________

Date: _____________________

Clinical rationale for why procedure is performed: Yes (2/2 pts)

Language describing what the procedure is (beyond the medical name) provided for the patient: Yes (2/2 pts)

Is it typed? Yes (1/1 pts)

Description of how procedure is performed provided: Yes (2/2 pts)

Is it typed? Yes (1/1 pts)

Is a quantitative probability provided for any procedure related risk: Yes (2/2 pts)

Is quantitative probability provided for risks: Yes (1/1 pts)

Is any benefit provided: Yes (2/2 pts)

Is any alternative provided: Yes (2/2 pts)

Date the form was shared with patient is provided: Yes (5/5 pts)

Total Score: 20/20

Supplementary Figure 1B: Consent for endoscopic lumbar discectomy for radiculopathy

Spatz et al. consent form abstraction and scoring system:

Clinical rationale for why procedure is performed: Yes (2/2 pts)

Language describing what the procedure is (beyond the medical name) provided for the patient: Yes (2/2 pts)

Is it typed? Yes (1/1 pts)

Description of how procedure is performed provided: Yes (2/2 pts)

Is it typed? Yes (1/1 pts)

Is a quantitative probability provided for any procedure related risk: Yes (2/2 pts)

Is quantitative probability provided for risks: Yes (1/1 pts)

Is any benefit provided: Yes (2/2 pts)

Is any alternative provided: Yes (2/2 pts)

Date the form was shared with patient is provided: Yes (5/5 pts)

Total Score: 20/20

Prompt:

Write a surgical consent form for L4-5 percutaneous endoscopic lumbar discectomy for a far lateral disc herniation causing an L4 radiculopathy. Include clinical rationale, describe the procedure, description of how the procedure is performed, quantitative and qualitative probability of risks, benefits, alternatives, and section to date and sign. Ensure that it is written at the average American reading level.

SURGERY PERMISSION FORM

Procedure: L4-5 Back Surgery for a Herniated Disc Causing Nerve Pain

Patient Name: ____
Date of Birth: __________________
Medical Record Number: ___________

Why You Need This Surgery:
You have a herniated disc in your lower back, causing nerve pain. This pain, weakness, and/or numbness hasn't gotten better with treatments like physical therapy, medication, and/or shots. The goal of the surgery is to help you feel better by taking out the herniated disc and freeing up the pinched nerve.

What the Surgery Is:
The L4-5 back surgery is a small surgery done with local numbing medicine and medicine to help you relax. The surgeon will make a small cut in your lower back and use a tube and a tiny camera to see inside. They'll use special tools to take out the herniated disc and free the pinched nerve. The cut will be closed with stitches or special tape.

How the Surgery Is Done:
You'll lie on your stomach on the operating table.
Your skin will be cleaned and sterilized.
Numbing medicine and medicine to help you relax will be given.
A small cut will be made in your lower back.
A tube and tiny camera will be put in to see inside.
The herniated disc will be removed, and the pinched nerve will be freed.
The cut will be closed with stitches or special tape.
You'll be watched in the recovery area until you're ready to leave.

Possible Risks:
Infection (<1% chance)
Bleeding (<1% chance)
Nerve injury (<1% chance)
Tear in the covering of the spinal cord (<1% chance)
Herniated disc coming back or not going away (10-15% chance)
Reaction to medicine (rare)
Not feeling completely better (varies)

Benefits:
Feeling better from pain, weakness, and/or numbness
Better quality of life
Small surgery with less damage to your body
Faster healing compared to bigger surgery

Other Options:
Keep trying treatments (medication, physical therapy, shots)
Open surgery to remove the disc

I, ____________________________, have read and understood the information above. I had the chance to ask questions, and all my questions have been answered. I agree to have the L4-5 back surgery as described above.

Patient Signature: ____________________________________ Date: ______________

Surgeon Signature: ____________________________________ Date: ______________

Supplementary Figure 1C: Consent for laparoscopic appendectomy for acute appendicitis

Spatz et al. consent form abstraction and scoring system:

Clinical rationale for why procedure is performed: Yes (2/2 pts)

Language describing what the procedure is (beyond the medical name) provided for the patient: Yes (2/2 pts)

Is it typed? Yes (1/1 pts)

Description of how procedure is performed provided: Yes (2/2 pts)

Is it typed? Yes (1/1 pts)

Is a quantitative probability provided for any procedure related risk: Yes (2/2 pts)

Is quantitative probability provided for risks: Yes (1/1 pts)

Is any benefit provided: Yes (2/2 pts)

Is any alternative provided: Yes (2/2 pts)

Date the form was shared with patient is provided: Yes (5/5 pts)

Total Score: 20/20

Prompt:

Write a surgical consent form for a laparoscopic appendectomy for acute appendicitis. Include clinical rationale, describe the procedure, description of how the procedure is performed, quantitative and qualitative probability of risks, benefits, alternatives, and section to date and sign. Ensure that it is written at the average American reading level.

[Header: Hospital / Clinic Name and Logo]
Surgery Permission Form
Appendix Removal for Swollen Appendix

Patient Name: ____
Date of Birth: ____
Medical Record Number: __________________________________

Why Surgery:

The patient has a swollen, pus-filled appendix. If not treated, this can cause serious problems like the appendix bursting, belly infection, and life-threatening infection harming organs. Removing the swollen appendix is the best way to stop these problems.

What Happens in Surgery:

To remove the appendix, the surgeon makes small cuts in the belly and uses a thin tube with a camera and light to see inside. They make three to four small cuts, fill the belly with gas, and take out the appendix with special tools. Risks: All surgeries have risks.

Some possible issues with this surgery include:

Infection: 3-5 out of 100 people
Bleeding: 1-2 out of 100 people
Harm to nearby organs (like intestines, bladder, blood vessels): 0.5-1 out of 100 people
Pus pocket: 2-5 out of 100 people
Problems with sleep medicine: less than 1 out of 100 people
Needing open surgery instead: 2-5 out of 100 people

Benefits:

No more swollen appendix symptoms
No risk of problems from a burst appendix
Shorter hospital stay than with open surgery
Less pain and faster recovery
Smaller scars

Other Choices:

Another option is open appendix removal with a bigger cut. Sometimes, people with mild swollen appendix can take antibiotics, but this might not work for everyone and could lead to more swollen appendix later.

Agreement: I, __________________________ (Patient Name), understand the information about the appendix removal surgery. I have talked with my surgeon about my condition, the treatment, and the risks and benefits. All my questions have been answered. I know there are no guarantees about how the surgery will go, and I choose to have the surgery.

Patient Signature: ______________________________ Date: _________________
Surgeon Signature: ______________________________ Date: _________________

Keep a copy of this signed form for your records.

Supplementary Figure 1D: Consent for coronary artery bypass grafting for acute NSTEMI

Spatz et al. consent form abstraction and scoring system:

Clinical rationale for why procedure is performed: Yes (2/2 pts)

Language describing what the procedure is (beyond the medical name) provided for the patient: Yes (2/2 pts)

Is it typed? Yes (1/1 pts)

Description of how procedure is performed provided: Yes (2/2 pts)

Is it typed? Yes (1/1 pts)

Is a quantitative probability provided for any procedure related risk: Yes (2/2 pts)

Is quantitative probability provided for risks: Yes (1/1 pts)

Is any benefit provided: Yes (2/2 pts)

Is any alternative provided: Yes (2/2 pts)

Date the form was shared with patient is provided: Yes (5/5 pts)

Total Score: 20/20

Prompt:

Write a surgical consent form for coronary artery bypass grafting for acute NSTEMI with multi-vessel coronary artery disease. Include clinical rationale, describe the procedure, description of how the procedure is performed, quantitative and qualitative probability of risks, benefits, alternatives, and section to date and sign. Ensure that it is written at the average American reading level.

HEART SURGERY PERMISSION FORM

Heart Bypass Surgery for a Serious Heart Condition

Patient Name: ______________________________ Date of Birth: ________________

Why You Need This Surgery:
You have a serious heart condition caused by blocked blood vessels in your heart. This makes it hard for your heart to get the blood and oxygen it needs. Treating this problem is important to prevent more damage to your heart and lower your chances of having a heart attack in the future.

What the Surgery Does:
Heart Bypass Surgery (CABG) is a surgery that helps blood flow better in your heart by going around the blocked blood vessels. The doctor will use a healthy blood vessel from your leg, arm, or chest to create a new path for blood to flow. This will help your heart get the blood it needs and lower your risk of having a heart attack in the future.

How the Surgery is Done:
You will be asleep during the surgery. The doctor will make a cut in your chest to reach your heart. If needed, a special machine might be used to help your heart and lungs work during the surgery. The doctor will attach the healthy blood vessel to the blocked one, making a new path for blood to flow. After this is done, the doctor will close the cut in your chest and watch your heart to make sure everything is okay before you go to the recovery room.

Risks and Chances of Them Happening:
All surgeries have risks. The risks and chances of them happening with this surgery are:
Infection (1-5%)
Bleeding (3-6%)
Stroke (1-2%)
Heart attack (3-5%)
Uneven heartbeat (10-30%)
Kidney failure (1-5%)
Death (1-3%)

Please remember that these chances are based on information from many people and might be different for you based on your health and other things.

Benefits:
The main benefits of this surgery are:
Less chest pain
Better blood flow to your heart
Lower risk of future heart attacks
Better quality of life and ability to do everyday activities

Other Options:
Other ways to treat your heart problem are:
Medicines and changes in your lifestyle
A procedure to open blocked blood vessels (PCI) with a small tube (stent)
Your doctor and heart specialist will talk about these options with you and suggest the best one for your situation.

Permission:

By signing below, I agree that I have read and understood the information in this form. I have talked with my healthcare provider about my heart problem, the surgery, and the risks and benefits. I know that I can say no to the surgery or change my mind at any time.

Patient Signature: ___________________________ Date: ____________________

Surgeon Signature: ___________________________ Date: ____________________

Supplementary Figure 1E: Consent for Mohs micrographic surgery for basal cell carcinoma

Spatz et al. consent form abstraction and scoring system:

Prompt:

Write a surgical consent form for Mohs micrographic surgery for basal cell carcinoma. Include clinical rationale, describe the procedure, description of how the procedure is performed, quantitative and qualitative probability of risks, benefits, alternatives, and section to date and sign. Ensure it is written at the average American reading level.

SKIN CANCER SURGERY PERMISSION FORM

Clinical rationale for why procedure is performed: Yes (2/2 pts)

Why Surgery is Needed:
Basal cell carcinoma (BCC) is a kind of skin cancer. Mohs surgery is a special way to remove BCC. It has the best chance of curing the cancer and leaves the least amount of scarring. This surgery is great for BCCs in areas that are important for looks or if they grow fast.

Language describing what the procedure is (beyond the medical name) provided for the patient: Yes (2/2 pts)

Is it typed? Yes (1/1 pts)

What Happens in Surgery:
Mohs surgery takes away the cancer and a thin layer of skin around it. Then, a doctor looks at the skin under a microscope to see if there are any cancer cells left. If there are, they take away more skin and check again. This continues until all the cancer is gone. This helps save healthy skin and lowers the chance of the cancer coming back.

Description of how procedure is performed provided: Yes (2/2 pts)

Is it typed? Yes (1/1 pts)

How Surgery is Done:
The surgery is usually done with numbing medicine at an outpatient office. The doctor takes out the cancer with a special tool. They also take a thin layer of skin around it and check it for cancer cells. If needed, more layers are removed and checked until all the cancer is gone. Then, the wound is closed with stitches, a skin flap, or a skin graft.

Is a quantitative probability provided for any procedure related risk: Yes (2/2 pts)

Is quantitative probability provided for risks: Yes (1/1 pts)

Risks of Surgery:
Infection (1-5%): Like any surgery, there's a chance of infection. Cleaning the wound and taking antibiotics can lower this risk.
Bleeding (1-2%): Heavy bleeding is rare but can happen. Usually, bleeding can be handled during the surgery.
Scarring (depends): Scars happen when skin heals. How big and noticeable they are depends on the tumor's size and where it was, and how the patient's skin heals.
Cancer coming back (1-5%): Even though Mohs surgery is the best treatment for BCC, there's a small chance it can come back.
Nerve damage (less than 1%): Sometimes, nerves near the surgery spot can be hurt, causing numbness or weakness that may last a short or long time.
Other risks: There might be other risks or problems with the surgery.

Is any benefit provided: Yes (2/2 pts)

Benefits:
High cure rate: Mohs surgery is the best way to treat BCC, so it lowers the chance of cancer coming back.
Saves healthy skin: Mohs surgery checks skin layers right away, so more healthy skin is saved. This leads to better looks and healing.

Is any alternative provided: Yes (2/2 pts)

Other Choices:
Regular surgery: The tumor is taken out with some healthy skin around it, but no microscope is used.
Freezing: The tumor is killed by freezing it with a very cold liquid.
Scraping and electric needle: The tumor is scraped away and the area is treated with an electric needle.
Medicine on the skin: Creams or ointments are put on the skin to kill the cancer cells.
Radiation therapy: X-rays are used to target and destroy the tumor.
No treatment: This might make the tumor grow more and cause more problems.

By signing, I say that I've read and understood this information. I agree to have Mohs surgery for basal cell carcinoma. I've talked about the surgery, risks, benefits, and other choices with my surgeon, and they've answered my questions. I know that there's no promise about how the surgery will turn out or how my body will react.

Date the form was shared with patient is provided: Yes (5/5 pts)

Patient Signature: ________________________________ Date: _______________

Surgeon Signature: ________________________________ Date: _______________

Note: Please make sure you've gone over this form with your surgeon and asked all your questions. You can change your mind and stop the surgery before it starts if you want to.

Total Score: 20/20

Legend

Supplementary Information

Generic Consent Forms at 15 Academic Medical Centers:

Copies of the 15 generic consent forms prior to and after undergoing LLM-facilitated simplification.

Supplementary Figure 1: Representative Examples of AI-Assisted Generation of New Surgical Consent Forms

Representative examples of use of GPT-4 to generate a new, procedure-specific surgical consent forms. The forms were annotated to demonstrate perfect 20/20 scores, based on the criteria developed for scoring consent form quality by Spatz *et al*.[14] All forms were generated at an average American reading level of 6th grade. **A:** Consent form for awake subthalamic nucleus deep brain stimulating electrode placement with microelectrode recording and test stimulation for Parkinson's disease; **B:** Consent form for a lumbar 4-5 percutaneous endoscopic lumbar discectomy for lumbar disc herniation causing an L4 radiculopathy; **C:** Consent form for laparoscopic appendectomy for acute appendicitis; **D:** Consent form for a coronary artery bypass grafting for acute non-ST segment elevation with multi-vessel coronary artery disease; **E:** Consent form for Mohs micrographic surgery for basal cell carcinoma. Relevant surgical experts (WFA, AET, NRS, NRS, and TJL, respectively) evaluated the forms and determined they conveyed appropriate information for the purposes of surgical consent.
